# New directions in male-tailored psychotherapy for depression

**DOI:** 10.3389/fpsyg.2023.1146078

**Published:** 2023-04-18

**Authors:** Lukas Eggenberger, Ulrike Ehlert, Andreas Walther

**Affiliations:** Department of Clinical Psychology and Psychotherapy, Psychological Institute, University of Zurich, Zurich, Switzerland

**Keywords:** depressive disorders, traditional masculinity ideologies, male depression, maletailored psychotherapy, treatment engagement, treatment effectiveness

## Abstract

**Purpose of review:**

Societal, cultural, and contextual norms about how men should be and behave (so called traditional masculinity ideologies; TMI) affect men’s presentation of depressive disorders, psychotherapy use, and treatment engagement. Only recently, however, male-tailored psychotherapy approaches for depressive disorders have been developed, which aim to systematically soften dysfunctional TMI. In this review, we outline the necessary groundwork as well as recent advances in research on TMI, men’s help-seeking, male depression, and their interrelatedness. Subsequently, we discuss the potential value of these findings for male-tailored psychotherapy for depressive disorders.

**Recent findings:**

A preliminary evaluation of a male-specific psychoeducation program found that a male-specific psychoeducation text could reduce negative affect as well as state shame and potentially elicit a shift from externalizing depression symptoms toward more prototypical depression symptoms. The *James’ Place* program, a male-tailored community-based service, improved suicidal men’s overall well-being, problems, functioning, and suicide risk. The *Heads Up Guys!* program, an eHealth resource aimed at depressed men, reported a high and increasing global interest in their website, with considerable visitor engagement. The *Man Therapy* online resource improved depressive symptoms, suicidal ideation, and help-seeking behavior. Finally, the *Men in Mind* program, an online training program for clinical practitioners, increased practitioners’ capacity to engage and support men in therapy.

**Summary:**

Male-tailored psychotherapy programs for depressive disorders, which are informed by recent advances in TMI research, may potentially increase therapeutic effectiveness, engagement, and adherence. While recent preliminary analyses of individual male-tailored treatment programs show promising results, extensive and systematic primary studies evaluating these programs are pending but greatly needed.

## Introduction

Depressed men, especially in comparison to depressed women, are often found to exhibit maladaptive and potentially harmful externalizing symptoms while also being reluctant to seek and engage in treatment (Seidler et al., 2016). Traditional sociocultural beliefs about the male gender role, which portray men as strong, being in control, and emotionally inexpressive, are thought to underlie these gender differences (Levant and Richmond, 2016). Such traditional beliefs about how men should be and behave directly conflict with the experience of certain depressive symptoms (e.g., depressed mood, loss of interest, or feelings of worthlessness) or seeking out professional help, which may be experienced as a form of losing control (Addis and Mahalik, 2003). These advancements led, on the one hand, to male-specific diagnostic instruments (Rice et al., 2013) as well as general guidelines for gender-related considerations for diagnosing depressed men (Rice et al., 2022). On the other hand, the potential interference of rigid beliefs about gender roles with psychotherapeutic processes led to the development and recommendation of male-specific treatment guidelines for mental health professionals who work with depressed men (American Psychological Association, 2018). Most recently, mental health programs specifically tailored to male patients have been developed, of which some preliminary reports show promising results for the treatment of depressed men.

In the following, we will first review the necessary groundwork as well as recent advances in research on TMI, men’s help-seeking, male depression, and their interrelatedness. Subsequently, we will discuss the potential value of these findings for male-tailored psychotherapy for MDD and report some preliminary findings from relevant male-tailored mental health programs.

### Identification of relevant literature

To identify relevant literature, we searched the five electronic databases *APA PsycNet, PSYINDEX, PubMed, SCOPUS,* and *Google Scholar* using the query “(*male-specific OR male-tailored*) *AND (psychotherapy OR intervention OR program) AND depression AND help-seeking AND traditional masculinity”* for the period of the last 2 years (2020–2022). This approach yielded a total of 63 search results, all of which were present in the *Google Scholar* search.

We subsequently filtered these results to include only (a) quantitative evaluations of novel intervention programs that (b) specifically aim to improve treatment of depression among men and that (c) draw on recent advancements in research on masculinity ideologies to increase their outreach and/or treatment effectiveness. During this step, an additional reference to the *James’ Place* program was found within the initial 63 search results, which fulfilled the inclusion criteria of the review but was not detected by our search query.

Overall, this led to the inclusion of the following five male-tailored mental health programs aiming to improve depression treatment in the present review article: *Male-Specific Psychotherapy Program for MDD*, *James’ Place*, *Heads Up Guys!*, *Man Therapy*, and *Men in Mind*.

### Traditional masculinity ideologies

Gender role ideologies generally refer to normative sociocultural beliefs about what constitutes a particular gender and its role in society (Thompson and Pleck, 1995), usually being classified on a continuum from more traditional or conservative to more egalitarian or liberal (Kroska, 2007). Thus, traditional beliefs about the male gender role – so called traditional masculinity ideologies (TMI) – are beliefs about how men should be and behave that predate the deconstruction of gender by second-wave feminism in the 1960s (Pleck, 1981, 1995). As such, TMI portray men as being in control, physically strong, and in contrast to stereotypically feminine traits and behaviors, such as being affectionate or expressing vulnerable feelings (Brannon and David, 1976; Levant and Richmond, 2016). As part of gender role socialization, boys learn and internalize TMI form an early age by receiving approval for gender-conforming behavior and punishment or disapproval for gender-non-conforming behavior (Vandello and Bosson, 2013). As a consequence, strict conformity to TMI often entails negative consequences because men may not live to their full potential out of fear of being socially sanctioned if not behaving according to TMI, which is referred to as gender role conflict (O’Neil, 2015).

Empirical research into men and masculinities revealed robust associations between strong TMI and negative mental health outcomes among men, particularly a reluctance to seek and accept help for mental health problems such as depression. For one, men with strong TMI are generally less likely to use psychotherapy than men with low TMI when experiencing comparable levels of depressive symptoms (Eggenberger et al., 2021, 2022). Especially TMI revolving around toughness and anti-femininity seem to negatively impact men’s use of mental health care services, for which the effect may be even stronger among depressed men (Sileo and Kershaw, 2020). However, strong TMI have also been linked to increased self-stigmatization of depressive disorders among men, which poses an additional barrier for depressed men to seek help from mental health professionals (Latalova et al., 2014; Walther and Seidler, 2020). And while an increase in help-seeking for mental health issues among women could be observed over a period of 12 years, help-seeking among psychologically distressed men seems to be stagnant (Brandstetter et al., 2017). However, TMI may not only hinder depressed men from seeking help, but they can also affect how men experience and express depressive symptoms (Seidler et al., 2016; Cavanagh et al., 2017).

### Male depression

Independent of sex, depressive disorders, including major depressive disorder (MDD), are the leading cause of global mental-health disease burden (Herrman et al., 2019; Global Burden of Disease Collaborative Network, 2021). According to the fifth edition of the *Diagnostic and Statistical Manual of Mental Disorders* (DSM-5; American Psychiatric Association, 2013), MDD is characterized by its cardinal symptoms of anhedonia (i.e., loss of interest/pleasure) and depressed mood, while also including additional symptoms such as feelings of worthlessness, excessive guilt, suicidal thoughts, fatigue, weight loss, psychomotor retardation, or concentration problems. Because some of these symptoms are in stark contrast with TMI that depict men as strong, invulnerable, and stoic (Addis and Mahalik, 2003; Levant and Richmond, 2016), some men tend to engage in avoidant behavior or mask these symptoms by presenting more externalizing symptoms, such as anger and aggression, substance abuse, or risk-taking (Nadeau et al., 2016; Oliffe et al., 2019; Seidler et al., 2019a). Particularly men with strong TMI express male-specific phenotypes of depressive disorders characterized by externalizing symptoms – also referred to as male or masculine depression (Magovcevic and Addis, 2005) – because they are more in line with TMI (Addis, 2008; Rice et al., 2019; Walther et al., 2021; McDermott et al., 2022). Thus, a large portion of depressed men may not be recognized by common diagnostic instruments that use more prototypical conceptualization of depressive disorders, such as the DSM-5 (Fields and Cochran, 2011). Undiagnosed depressed men consequently do not receive the professional support or treatment they need, which increases their risk to engage in maladaptive and harmful behaviors (e.g., alcohol abuse or aggressive behavior) as a way to self-manage their depressive symptoms (Rutz and Rihmer, 2009; Seidler et al., 2016).

Furthermore, externalizing symptoms as a phenotypic feature of male depression bear great clinical importance, as they have been linked to increased suicidal ideation and suicide risk among men (Coleman, 2015; Oliffe et al., 2019; Zajac et al., 2020). However, it remains less clear whether externalizing symptoms themselves (e.g., engaging in risky behavior) or underlying TMI increase men’s risk for suicidality. For example, a study by Coleman et al. (2020) found men with strong TMI to be more than twice as likely to die by suicide than men with low TMI, while also being less likely to disclose suicidal ideation. Concordantly, Walther et al. (2022) found men with strong TMI who experienced status loss – status being a core dimension in many conceptualizations of TMI – to be about four times more likely to have attempted suicide in the past month. Particularly masculinity norms revolving around stoicism, self-reliance, and verbal aggression have been found to increase suicidal desire among men who experience psychological distress (Daruwala et al., 2021).

Male depression and its externalizing symptoms have been broadly recognized, be it by developing male-specific depression diagnosis instruments, such as the MDRS-22 (Rice et al., 2013), or by highlighting the importance of considering potential gender differences when diagnosing MDD in the most recent text revision of the DSM-5 (Rice et al., 2022). Notably, the gender gap in depressive disorders (i.e., men being half as likely to be diagnosed with depressive disorders than women; Salk et al., 2017; Vos et al., 2017) disappears when externalizing symptoms are considered for depression diagnoses (Martin et al., 2013; Cavanagh et al., 2017). As a next essential step, however, these findings related to gender differences, TMI, and their relation to help-seeking and externalizing depressive symptoms need to be integrated into mental health treatment and psychotherapy programs for depressed men (Brandstetter et al., 2017).

### Male-tailored psychotherapy

While cognitive behavioral therapy (CBT) is an effective treatment for depression (e.g., Munder et al., 2019), conflicting findings exists whether men and women benefit equally from CBT as a depression treatment. For one, meta-analytic findings suggest that there seem to be no differences between men and women regarding the effectiveness of CBT for MDD (Cuijpers et al., 2014) nor in the individual dropout rates in psychotherapy programs for MDD (Cooper and Conklin, 2015). However, many primary studies suggest that male gender predicts worse outcomes in group or internet-based CBT for MDD (Ogrodniczuk, 2007; Donker et al., 2013), lower expectations regarding CBT for MDD (Vîslă et al., 2019), higher dropout rates (Pederson and Vogel, 2007; Simon and Ludman, 2010), as well as lower therapeutic alliance in couples therapy (Bartle-Haring et al., 2012; Halford et al., 2016). Such mixed findings could, however, be due to the interindividual variability in the conceptualization, endorsement of, and conformity to TMI among men rather than associated with the binary gender classification (i.e., being male or female) used in the meta-analyses by Cuijpers et al. (2014) and Cooper and Conklin (2015).

For example, many core concepts of TMI, such as being in control or emotional inexpressiveness (Levant and Richmond, 2016), potentially conflict with therapeutic processes relevant for effective treatment outcomes. Using psychotherapy, as a form of accepting external support, means giving up individual control to a certain extent (Englar-Carlson and Kiselica, 2013). Similarly, effectiveness of therapeutic processes greatly depends on patients opening up and accepting and showing personal vulnerabilities (Good et al., 2005). As a consequence, male-tailored psychotherapy programs should consider and create awareness of the patient’s and clinician’s gender role socialization as well as its impact on male-specific depression symptomatology and help-seeking behavior (Seidler et al., 2019b). Thus, despite mixed findings regarding gender differences in psychotherapy effectiveness, recommendations for tailored counseling programs for men –particularly those with strong TMI –have been elaborated by scholars and clinicians working with depressed men (Mahalik, 1999; Englar-Carlson and Kiselica, 2013; O’Neil, 2013; American Psychological Association, 2018; Seidler et al., 2018, 2019b).

To date, however, very limited findings exist regarding the evaluation of male-tailored and gender role socialization-oriented psychotherapy approaches. Primack et al. (2010) qualitatively reported positive feedback of six male patients toward a male-tailored group psychotherapy for MDD. Nahon and Lander (2010), on the other hand, found no specific effect of gender role re-assessment on recently divorced male patients’ experience of gender role conflict, emotional expressiveness, or psychological well-being. While such qualitative findings call for further research on the effectiveness of male-tailored psychotherapeutic intervention programs, some promising reports from recently established male-tailored mental health programs for depressed men have emerged in the past few years.

In an online randomized controlled investigation for a male-tailored psychoeducation for MDD, Walther and Eggenberger (2022) examined 152 men who experienced psychological distress (2022). Therein, one group of men received standard CBT-based psychoeducation for MDD and the other group received a male-tailored psychoeducation for MDD, which included information on the gender-specific etiology of depressive symptoms, gender role socialization, and TMI. Men who received the male-tailored psychoeducation showed a greater reduction in negative affect, state shame, and a potential shift from externalizing toward more prototypical depressive symptoms as compared to men who received a standard CBT-based psychoeducation. Building on these findings and incorporating further insights from research on male depression, our research group developed a male-specific psychotherapy program for depressed men based on CBT principles, which is currently being evaluated in a randomized controlled trial (Walther et al., 2023). The primary aim is to evaluate the potential superior effectiveness, efficacy and reduced therapy drop-out of this male-specific psychotherapy program (MSPP) for MDD in depressed men compared with CBT for MDD and a wait list contact control group. However, trial results are expected to be available earliest in 2026.

An internal report of the *James’ Place* model, a community-based service delivering professional clinical interventions for suicidal men in the United Kingdom (Hanlon et al., 2022), showed a positive improvement of clinical outcome measures (i.e., well-being, problems, functioning, and risk) as well as qualitative findings of increased hope, improved close relationships, and reduced suicidal thoughts among men after completion of the program (Saini et al., 2020).

Ogrodniczuk et al. (2021) recently reported insights into the web analytics of the *Heads Up Guys!* program, an eHealth resource aimed at depressed men that provides information on men’s mental health, depression treatment and care, as well as suicide prevention strategies (Ogrodniczuk et al., 2018). They found not just a generally high and increasing (over 5 years) global interest in their website, but also considerable visitor engagement. *Man Therapy*, another online intervention program for men primarily based on CBT-concepts, has been found to improve men’s depressive symptoms, suicidal ideation, and professional help-seeking behavior in two individual randomized-controlled trials (Frey et al., 2022; Gilgoff et al., 2022). Importantly, the “dosing” or amount of engagement with *Man Therapy* and whether other interventions were used simultaneously has so far not been accounted for. Seidler et al. (2022) conducted a pilot evaluation of the *Men in Mind* program, an online training program for clinical practitioners providing psychotherapy for men. After completion, clinicians reported a significant increase in their capacity to engage and support men in therapy, of which the effect was even stronger among female clinicians than male clinicians.

### Conclusion

Depressed men, particularly those with strong TMI, represent a challenging patient population. They are not only more reluctant to seek and engage in treatment, but also often present with maladaptive and potentially harmful externalizing depressive symptoms that are more in line with TMI. Recent advances in TMI research may therefore not only improve the diagnostic process for depressed men with phenotypically externalizing symptoms, but male-tailored psychotherapy programs for depression could also potentially increase therapeutic effectiveness, engagement, and adherence. Specifically, tailored treatment approaches need to consider and potentially soften rigid traditional gender role beliefs in an attempt to avoid interference with psychotherapeutic processes. While recent preliminary analyses of individual male-tailored mental health programs show promising results for the treatment of depressed men, extensive and systematic primary studies evaluating these male-tailored psychotherapy programs are pending but greatly needed.

## Author contributions

LE and AW: conceptualization and methodology. LE: writing—original draft preparation. AW and UE: writing—review and editing, funding acquisition, resources and supervision. All authors contributed to the article and approved the submitted version.

## Funding

AW and LE were supported by a grant from the Swiss National Science Foundation awarded to AW (PZPGP1_201757). Research infrastructure and materials to support the research were provided by UE from internal funds of the University of Zurich.

## Conflict of interest

The authors declare that the research was conducted in the absence of any commercial or financial relationships that could be construed as a potential conflict of interest.

## Publisher’s note

All claims expressed in this article are solely those of the authors and do not necessarily represent those of their affiliated organizations, or those of the publisher, the editors and the reviewers. Any product that may be evaluated in this article, or claim that may be made by its manufacturer, is not guaranteed or endorsed by the publisher.
